# Nearly Missed: Painless Aortic Dissection Masquerading as Infective Endocarditis

**DOI:** 10.7759/cureus.2587

**Published:** 2018-05-07

**Authors:** Sukhdeep Bhogal, Muhammad Khalid, Ghulam Murtaza, Tarun Bhandari, Jeffrey Summers

**Affiliations:** 1 Department of Internal Medicine, Quillen College of Medicine, East Tennessee State University, Johnson City, USA; 2 Department of Internal Medicine, East Tennessee State University, Quillen College of Medicine, Johnson City, USA

**Keywords:** acute aortic dissection, painless aortic dissection, infective endocarditis

## Abstract

Aortic dissection is a life-threatening emergency associated with significant mortality rate. Early diagnosis is essential to improve the survival. Although the most common presentation is severe chest pain, it can be variable leading to delay in the diagnosis especially if it is painless. Painless aortic dissection is a rare entity with sparse data available based on case reports. We present a case of a young male with an atypical presentation where the presumptive diagnosis of infective endocarditis was made based on initial presentation but was eventually diagnosed as painless aortic dissection.

## Introduction

Aortic dissection (AD) is a life-threatening condition that requires an early diagnosis to prevent mortality. The most typical presentation is severe chest pain. The estimated incidence ranges from 2.5 to 3.5 per 100,000 person-years. Painless aortic dissection is a relatively rare presentation of aortic dissection, an entity that can be easily missed because of atypical presentation. A high index of suspicion is required for diagnosis in these cases. We herein present a case of painless aortic dissection, where the presumptive diagnosis of infective endocarditis was made based on initial presentation but was eventually diagnosed as painless AD.

## Case presentation

A 43-year-old Caucasian male with a medical history significant for intravenous (IV) drug use presented to the emergency department with restlessness, agitation, and profuse sweating. He reported no pain in the chest, neck, back, or abdomen. He denied a history of diabetes mellitus, hypertension, coronary artery disease, or connective tissue disease and was not taking any medication. He reported smoking one pack per day for the last 20 years. On presentation, he had a blood pressure of 120/32 mmHg; pulse of 90 beats per min; temperature of 100.1 F, and respiratory rate of 24 breaths/min. His oxygen saturation was 95% on two-liter of oxygen. He was fully oriented but found to be agitated and restless. A cardiovascular examination revealed grade 3/6 decrescendo diastolic murmur heard best on the left parasternal border on expiration. There were clear breath sounds bilaterally with no audible wheezes or crackles. The abdominal and neurological exams were benign.

**Figure 1:**
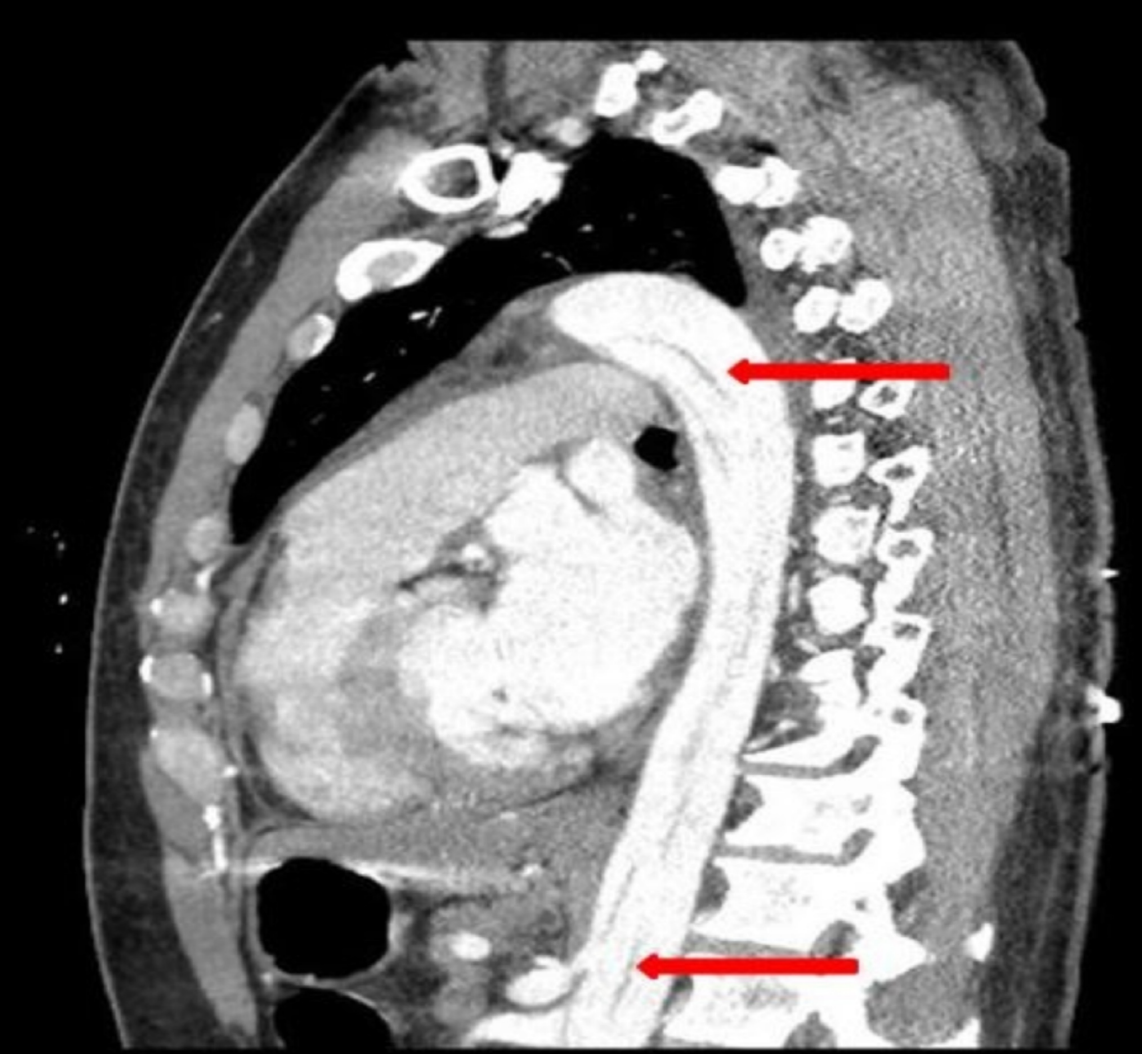
Computed tomography scan showing evidence of dissection in the aortic arch (upper arrow) and extension into the abdominal aorta (lower arrow).

**Figure 2:**
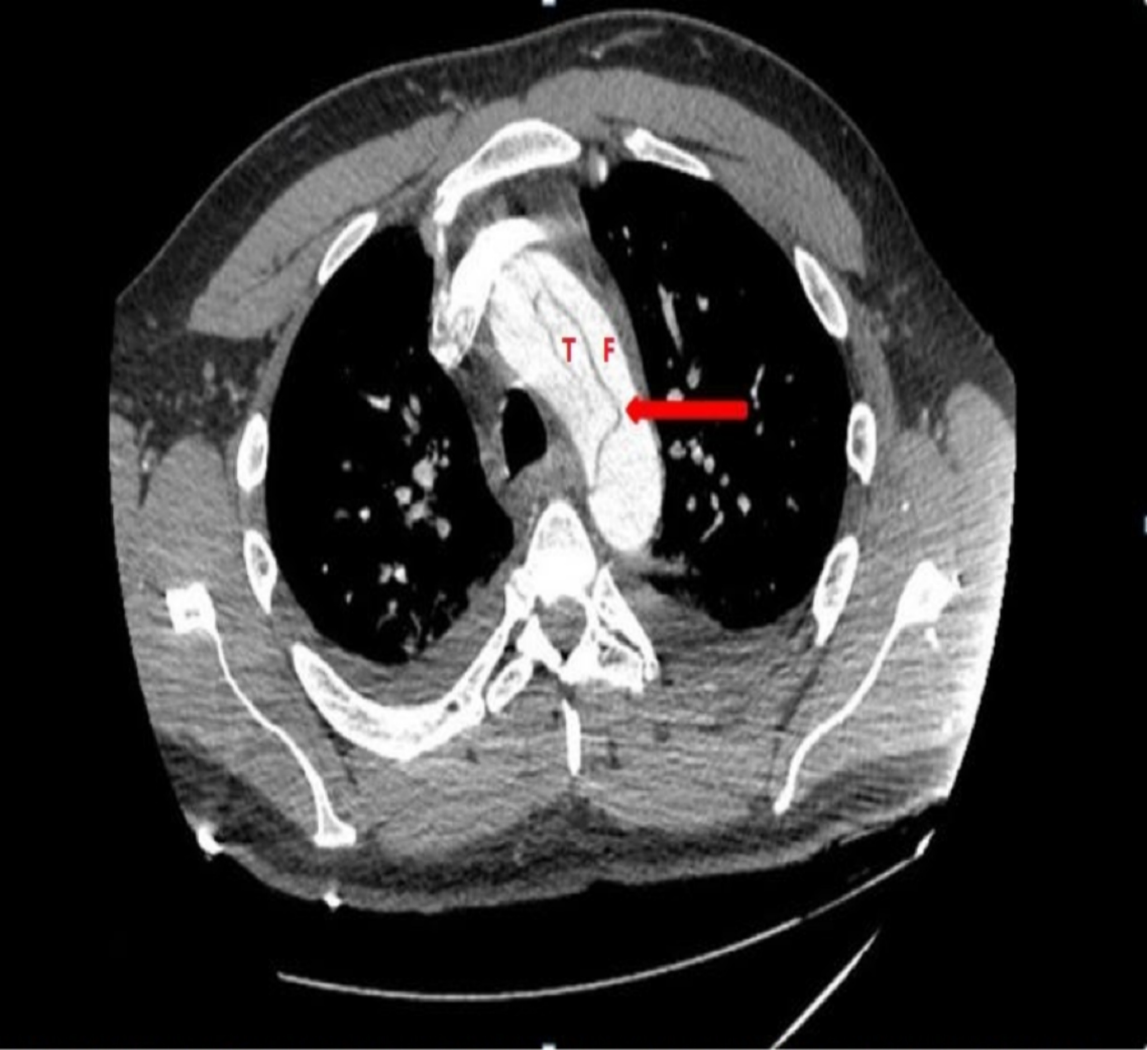
Computed tomography showing aortic dissection with arrows pointing towards the intimal tear in the aortic arch separating the true lumen (T) from the false lumen (F).

**Figure 3:**
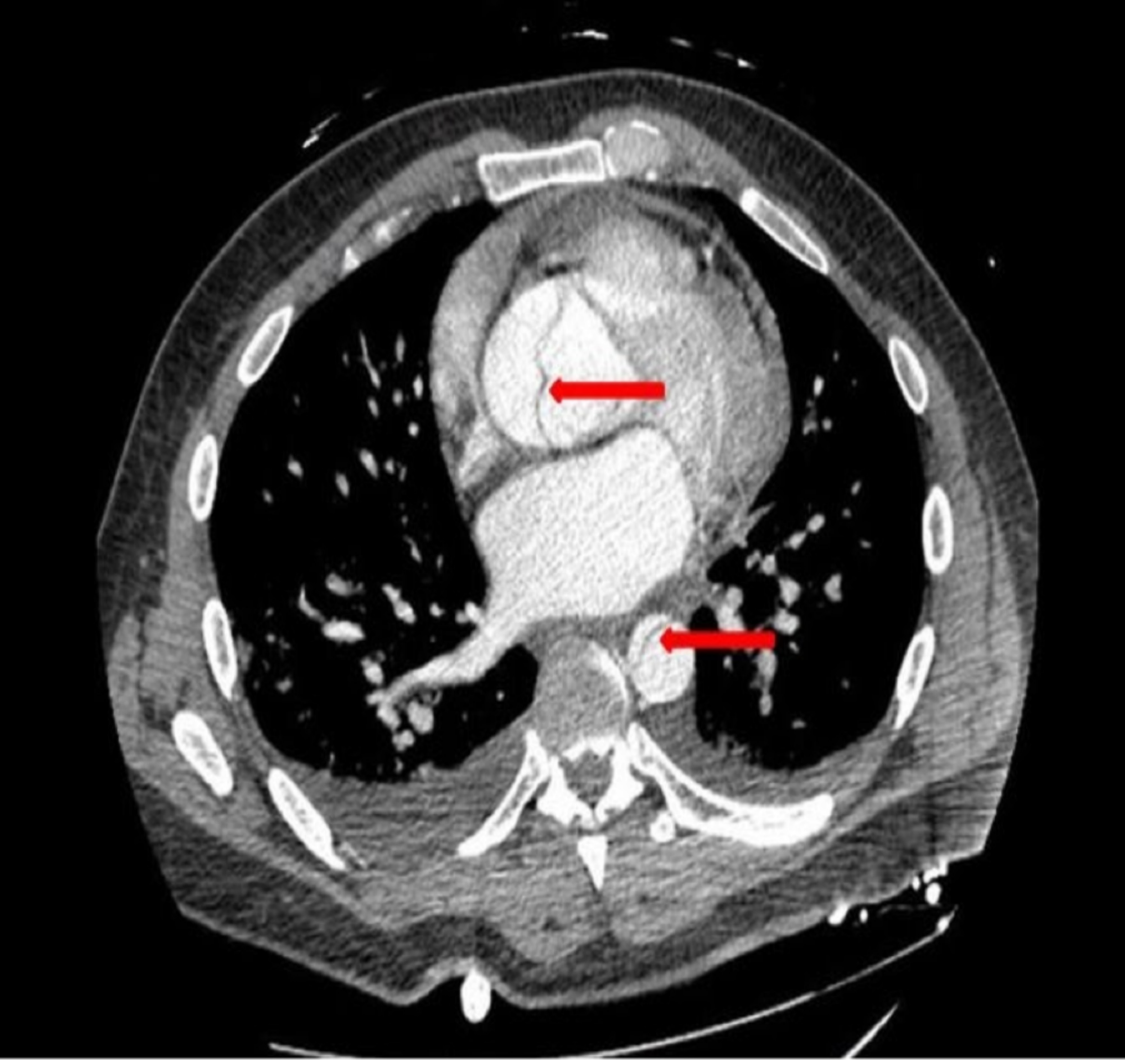
Computed tomography showing evidence of dissection in the ascending and descending aorta as shown by arrows.

His electrocardiogram showed nonspecific T wave changes in V1-V2 with sinus rhythm. His chest X-ray was unremarkable as well. The laboratory reports were normal except for creatinine of 2.5 mg/dl. Based on the history of IV drug abuse and auscultation consistent with a murmur and low-grade fever, the presumptive diagnosis of infective endocarditis was made. Blood cultures were drawn and empiric broad-spectrum antibiotics were started. A transthoracic echocardiogram (TTE) was ordered to look for possible valvular pathology/vegetation. TTE showed aortic root dilatation and aortic insufficiency along with the possibility of dissection in the ascending aorta. A computed tomography (CT) angiogram was ordered emergently that revealed aortic dissection involving the ascending aorta, arch (Figures 1-2) with an aneurysm measuring up to 5.5 cm, extending into the descending aorta (Figures 1-3). Emergent aortic root replacement along with ascending aorta and hemi-arch replacement were performed. The postoperative course was uneventful and he was discharged in a stable condition.

## Discussion

Aortic dissection (AD) is a life-threatening emergency associated with significant mortality rate. Early diagnosis is essential to improve the survival. It is subcategorized as type A involving the ascending aorta and type B involving the aorta distal to the left subclavian artery. The estimated incidence ranges from three to four per 100,000 person-years [1]. A review conducted by the International Registry of Acute Aortic Dissection (IRAD) revealed that two-thirds of the patients presenting with AD are men with average age ranging from 62 ± 14 years and 64 ± 14 years for type A and B AD, respectively [2]. With recent advances and increased awareness, there has been a significant decrease in mortality of type A AD from approximately 31 to 22% [2]. However, the trend of the mortality rate of type B AD remains unchanged from 12-14% [2].

The most important predisposing factor for AD is hypertension [2, 3]. Marfan syndrome, atherosclerosis, bicuspid aortic valve or large aortic diameter are other important predisposing factors particularly in young patients [4]. The pathophysiology involves medial degenerative changes either via inflammatory or non-inflammatory process, leading to disruption and loss of smooth muscle and elastic fibres in the aortic media [5]. Also, increased expression of matrix metalloproteinases (MMP) especially MMP-2 and MMP-9 in the media of aortic aneurysms have been found and are known to exhibit elastolytic activity. Subsequently, medial degeneration causes weakening of the aortic wall and increased intraluminal pressure results in the aortic aneurysm and eventually rupture or dissection. Intimal tear results in the entering of blood into the medial layer of the aortic wall, forming a dissection flap separating true lumen and false lumen, as seen in our case (Figure 2). Furthermore, it can propagate either anterogradely or retrogradely involving the heart or abdominal vessels. Consequently, various complications such as aortic insufficiency, congestive heart failure, myocardial infarction, pericardial effusion or tamponade, syncope, stroke, pleural effusion, and mesenteric ischemia are associated with acute dissection [5].

The most common classic presenting symptom is sudden onset, severe chest pain, which is seen in more than 80% of cases in type A and 70% of cases of type B AD [2]. The presentation can be variable leading to a delay in the diagnosis especially if it is painless. Painless AD is a rare entity, with sparse data available based on case reports presented as paraplegia [6, 7]. A study involving 977 patients revealed that about 63 (6.4%) patients had painless AD and these patients were found to have a higher incidence of syncope, congestive heart failure, and stroke relative to classic painful AD patients [8]. On the contrary side, our patient did not have the aforementioned presentation of painless AD. Auscultated murmur of aortic insufficiency has been reported in almost 32% cases on presentation [3], as seen in our case.

Recent IRAD data suggests that in patients with AD reports of normal chest X-ray on presentation has significantly increased [2], similar to our case. Transthoracic echocardiography (TTE) is a rapid bedside tool of assessment for suspected acute cardiac complications such as pericardial tamponade, aortic insufficiency, and regional wall motion abnormalities [9]. Further diagnostic modalities include magnetic resonance (MR) angiography, computed tomographic (CT) angiography, and transesophageal echocardiography (TEE) with reported sensitivities of almost 98, 98, and 97, respectively [10]. TEE is the diagnostic imaging test of choice particularly in cases of hemodynamic compromise [10]. The data suggests that the frequency of CT scan has been increased from 46% to more than 70% in the last 17 years for the diagnosis of type A AD, while there has been no change in frequency in type B AD [2]. In contrast, the use of TEE has been decreased to half for the diagnosis of type A AD [2].

Management is based on the concept of reducing the force of left ventricular ejection and controlling blood pressure, which are the major determinant factors for the extension of acute dissection. Beta blockers are the cornerstone of the management plan with the goal to achieve blood pressure between 100-120 mmHg and heart rate less than 60 beats per minute [9]. Verapamil or diltiazem can be used as a substitute particularly if the patient is intolerant to beta blockers. If beta blockers are insufficient to control blood pressure, a vasodilator such as sodium nitroprusside is recommended [9]. The definitive treatment for type A dissections requires emergent surgery, which includes resection of the aneurysmal aorta and replacement of the ascending aorta [5]. Also, aortic valve replacement is essential, if it is not feasible to restore the competency of the aortic valve via resuspension [5]. The significant reduction in mortality rate of type A AD is attributable to the reduction of surgical mortality rate from 25 to 18% [2]. Type B dissections are managed with medical therapy unless associated with complications such as aortic expansion, progression of dissection, end-organ malperfusion syndromes, or persistent pain which warrants endovascular interventions [9].

## Conclusions

Painless aortic dissection is a relatively rare presentation of aortic dissection. Our case stresses the importance of early diagnosis, particularly in atypical presentations, which could be invaluable in improving survival outcomes.
